# Oxidant stress and renal function among children with chronic kidney disease: a repeated measures study

**DOI:** 10.1038/s41598-020-59962-9

**Published:** 2020-02-21

**Authors:** Melanie H. Jacobson, Mengling Liu, Yinxiang Wu, Susan Furth, Bradley Warady, Howard Trachtman, Leonardo Trasande

**Affiliations:** 1 Department of Pediatrics, Division of Environmental Pediatrics, NYU Langone Medical Center, New York, NY USA; 2 Departments of Population Health and Environmental Medicine, NYU Langone Medical Center, New York, NY USA; 3 Division of Nephrology, Department of Pediatrics, Children’s Hospital of Philadelphia, Philadelphia, PA USA; 4 Division of Nephrology, Department of Pediatrics, Children’s Mercy Kansas City, Kansas City, MO USA; 5 Department of Pediatrics, Division of Nephrology, NYU Langone Medical Center, New York, NY USA; 6 NYU Wagner School of Public Service, New York, NY USA; 7 NYU College of Global Public Health, New York, NY USA

**Keywords:** Medical research, Nephrology

## Abstract

It is hypothesized that chronic kidney disease (CKD) induces oxidant stress which contributes to the decline in kidney function. However, few studies have incorporated longitudinal designs and no studies have investigated this association among children. Using data from the Chronic Kidney Disease in Children (CKiD) study, we examined longitudinal associations between urinary biomarkers of oxidant stress, 8-OH deoxyguanosine (8-OHdG) and F2-isoprostane, and measures of renal function and blood pressure among children with CKD. Baseline levels of 8-OHdG were positively associated with estimated glomerular filtration rate (eGFR) over time and a log-unit increase in baseline 8-OHdG predicted a 5.68 ml/min/1.73 m^2^ increase in eGFR (95% Confidence Interval (CI): 3.75, 7.61). This association was attenuated when longitudinal measures of 8-OHdG were analyzed in relation to longitudinal eGFR (per log-unit increase in 8-OHdG, β = 0.81, 95% CI: 0.22, 1.39). Baseline 8-OHdG concentrations were also associated with decreased proteinuria over time, as measured by urinary protein:creatinine ratio. In addition, F2-isoprostane concentrations were associated with increases in eGFR, but only when baseline levels (vs. longitudinal levels) were considered in relation to longitudinal eGFR. There were no significant associations between either 8-OHdG or F2-isoprostane and blood pressure over time. Urinary measures of oxidant stress are not associated with worsening GFR over time. Our findings suggest that excretion of these biomarkers may be influenced by changes in glomerular and tubular function in varying patterns, which would limit their value in evaluating the impact of oxidant stress on CKD progression in children.

## Introduction

Chronic kidney disease (CKD) is a progressive condition that ultimately leads to loss of kidney function and a need for renal replacement therapy (RRT), either dialysis or transplant^1^. CKD is common in the US, affecting 11% of adults^2,3^, and is projected to increase over time^4^. Although substantially less common in children, it is associated with substantial adverse sequelae specific to this age group, such as growth impairment, cognitive deficits, and cardiovascular complications^5,6^.

Several factors have been shown to affect the rate of decline in kidney function among those with CKD^7^. Mechanistically, it has been widely hypothesized that oxidant stress accelerates kidney function decline^8–10^. This can arise within the glomeruli or the tubulointerstitium, in the later compartment secondary to increased tubular workload and oxygen consumption^11,12^. An imbalance in the production of reactive oxygen species and antioxidant defenses disturbs cell signaling and promotes injury to the kidneys, limits cellular repair mechanisms, and accelerates functional decline. In several cross-sectional studies, plasma levels of oxidant stress have been found to be associated with advanced stages of CKD^13–15^. Further evidence in support of this relationship is limited by a lack of longitudinal studies examining both oxidant stress and kidney function over time.

F_2_-isoprostane, an end-product of lipid peroxidation and 8-OH deoxyguanosine (8-OHdG), a measure of DNA damage, are two widely used biomarkers of systemic oxidant stress^9,16–19^. Although these markers have been studied in adult populations, no studies to our knowledge have evaluated their potential contribution to CKD among children. To better understand whether oxidant stress impacts kidney function among children with CKD over time, we utilized data and biospecimens collected by the Chronic Kidney Disease in Children (CKiD) study, a large longitudinal cohort study of children with CKD.

The purpose of this study was to examine the longitudinal associations between urinary biomarkers of oxidant stress, F2-isoprostane and 8-OHdG, and measures of renal function among children with CKD. First, we estimated associations between F2-isoprostane and 8-OHdG at baseline and measures of renal function over time: estimated glomerular filtration rate (eGFR) and proteinuria (as measured by urinary protein to creatinine ratio (UPCR)); systolic and diastolic blood pressure (SBP and DBP, respectively) over time; and renal outcomes: time to end-stage kidney disease (ESKD) and/or RRT. Second, we estimated the associations between longitudinally measured levels of urinary F2-isoprostane and 8-OHdG in relation to these same outcomes measured over time. Lastly, in both analyses, we examined whether the associations varied over time.

## Methods

### Data collection

The CKiD study is a multi-centered prospective cohort study of children aged 6 months to 16 years with mild-to-moderate CKD with the overall goal of identifying predictors and sequelae of CKD progression. The CKiD study procedures and protocol has been previously described^20^. Briefly, children receiving care for CKD were recruited at sites throughout the US and Canada and participated in annual data collection study visits until the initiation of RRT or ESKD. Annual study visits included a questionnaire, a physical examination conducted by study staff, and biological specimen collection (i.e., serum, plasma, and urine samples). Biological specimens were collected between 2005 and 2014 and were stored at −80 °C in a central biorepository for future use in ancillary studies.

This study utilized data from CKiD starting at least 3–6 months after the baseline visit. Clinical, biospecimen, and survey data from each study visit were used for exposure, outcome, and covariate data.

The Institutional Review Board at each CKiD study site approved the study protocol and all research was performed in accordance with established guidelines. Informed consent was obtained from all parents or legal guardians and assent from all participants depending on their age and institutional guidelines. The New York University School of Medicine Institutional Review Board deemed this project exempt from review due to data collection being complete and the dataset de-identified.

### Measures

#### Oxidant stress

Urine samples with sufficient volume were analyzed for 8-OHdG and F2-isoprostane at the NYU High Throughput Biology Laboratory (N = 2,464 samples; N = 618 individuals and N = 1,286 samples; N = 522 individuals, respectively). 8-OHdG was quantified by competitive ELISA using the OxiSelect™ Oxidant DNA Damage ELISA (Cell Biolabs, Inc., San Diego, CA). Similarly, F2-isoprostane was measured with a competitive enzyme-linked immunoassay, the OxiSelect™ 8-iso-Prostaglandin F2α ELISA kit. All analyses were conducted in duplicate as directed by the manufacturer. Intra-assay coefficients of variation (CV) ranged from 4.6–11.1% for 8-OHdG and 6.8–9.7% for F_2_-isoprostane. Inter-assay CVs ranged from 3.9–16.6% for 8-OHdG and 14.2–15.9% for F_2_-isoprostane. Measures below the limit of detection (LOD) were imputed by the LOD divided by the square root of 2^21^.

Urinary creatinine was measured in first morning urine samples to account for dilution in the central laboratory (University of Rochester) for CKiD^22^.

#### Outcomes

Several measures of renal function were examined. The primary outcome was eGFR, calculated using the modified Schwartz equation^23^: eGFR (ml/min/1.73 m^2^) = 0.413 × height (cm)/serum creatinine.

Other outcome measures of interest included UPCR,SBP, DBP, and time to ESKD/RRT. Methods for the measurement of these outcomes in the CKiD study have been previously described. All laboratory measures were conducted at the central CKiD laboratory (University of Rochester)^22,24^. Briefly, total urine protein was determined in first morning samples using an immunoturbidimetric assay and UPCR was calculated as the ratio of total urinary protein to urinary creatinine (mg:mg). SBP and DBP were measured in the right arm by auscultation using an aneroid sphygmomanometer. Three measurements at 30-second intervals were measured and the average of the three readings was taken. Blood pressure measures were standardized to z-scores according to the National High Blood Pressure Education Program Fourth Report^25^. ESKD status or initiation of RRT was determined by the start of dialysis or transplantation^26^.

### Statistical analysis

The distributions of 8-OHdG and F2-isoprostane were explored in univariate analyses as creatinine-corrected measures (ng/mg Cr). Differences between strata of participant characteristics were compared and tested using linear mixed models (LMM) with log-transformed oxidant stress biomarkers and subject-specific random intercepts. Predicted means and 95% confidence intervals were output.

In order to estimate the associations between oxidant stress biomarkers and correlates of renal function over time, LMMs were fit with subject-specific random intercepts to account for the within-individual correlation among repeated measures over time and cross-subject heterogeneity as baseline. Two sets of models were considered. First, models were fit with baseline oxidant stress measures and longitudinal outcomes repeated over time. Second, models were fit with longitudinal oxidant stress measures and all outcomes over time. Each model was fit with a single oxidant stress measure (i.e., 8-OHdG or F2-isoprostane) and a single outcome (i.e., eGFR, UPCR, SBP, or DBP).

In order to evaluate associations between oxidant stress measures and time to RRT, Cox proportional hazards models were fit. As with the LMMs for other renal outcomes, two sets of models were tested. First, baseline 8-OHdG or F_2_-isoprostane were examined in relation to time to RRT, and second, longitudinal or time-varying 8-OHdG or F_2_-isoprostane were examined using extended Cox models^27^. Time at risk started at the baseline visit and continued until the initiation of RRT or censorship at the end of follow-up. The proportional hazards assumption was checked for all covariates using visual inspection of log-log survival curves and testing of Schoenfeld residuals and covariate-by-time interaction terms.

Due to the right skewed distributions of 8-OHdG and F2-isoprostane, they were natural log-transformed in order to reduce the influence of extreme values. Models were fit with 8-OHdG and F2-isoprostane expressed on the volume basis (ng/ml) and creatinine was controlled for as a covariate instead of indexing the oxidant stress measure by creatinine (ng/mg Cr) in order to separate any impacts of oxidant stress and creatinine^28^, and because the same creatinine measure was included in one of the primary outcomes of interest (i.e., UPCR), which could induce a spurious correlation due to urinary creatinine associations rather than with oxidant stress itself^29^. UPCR was also natural-log transformed due to its skew in order to approximate a normal distribution. After transforming UPCR, raw and conditional residuals from the LMMs were normally distributed. Covariates in the LMMs were selected a priori based on the literature^2,7,13,22,26,30–35^. All models were adjusted for study visit, urinary creatinine, sex, race/ethnicity, age, glomerular disease type, urinary cotinine, and BMI Z-scores. Additionally, in models for eGFR and UPCR, blood pressure was controlled for; in models for blood pressure, antihypertensive medications were controlled for. Lastly, we examined whether associations varied over time by including an interaction (i.e., cross-product term) between study visit and the respective oxidant stress biomarkers.

We conducted several sensitivity analyses. First, because creatinine is used both as a factor to account for urinary dilution and is itself affected by kidney tubule processing^36^; as well as being contained in one of the outcomes of interest, UPCR, we applied an alternative method for adjusting for urinary concentrations. We calculated estimated creatinine excretion rate (eCER) and controlled for it in all models instead of urinary creatinine^29,37,38^. Second, in order to preserve the temporality of exposure preceding the outcome, we fit all models again with baseline oxidant stress measures as the exposures and the same outcomes over time, but starting at the second visit. Third, we stratified all analyses by eGFR levels at baseline and again by median UPCR levels at baseline. Separate models were run among those with eGFR levels ≥45 ml/min/1.73 m^2^ (CKD Stage 3a and higher) and <45 ml/min/1.73 m^2^ (CKD stage G3b and lower)^39^ in order to assess whether urinary excretion of oxidant stress markers could be altered among those with substantially depressed kidney function (i.e., eGFR levels < 45 ml/min/1.73 m^2^ at baseline), thus affecting the observed associations. In addition, separate models were run among those with UPCR ≥ 0.30 mg/dL (i.e., the median) and among those with UPCR < 0.30 mg/dL: mg/dL. Finally, because the CKiD study followed children until the initiation of renal replacement therapy or ESRD, drop-out was, by definition, informative. In order to assess whether this affected results, we created a balanced dataset, in which each individual contributed just the first three observations. We then re-ran all models using this dataset.

All analyses were performed using SAS Version 9.4 (Cary, NC). Associations were evaluated by statistical significance at α = 0.05.

## Results

The study population consisted of 618 children contributing 2,464 observations (mean = 4.0 visits per child over time, standard deviation (SD) = 1.6) over a mean of 2.9 years (SD = 1.6). The majority of children (79.9%) had at least three visits over time. Most were male (63.8%), White (58.3%), and had non-glomerular CKD (89.2%) (Table 1). The mean age at the start of follow-up was 10.8 years (SD = 4.4). At the initial study visit, most children had moderate CKD. The average eGFR was 51.9 ml/min/1.73 m^2^ (SD = 20.0), and only 14.2% had eGFR less than 30 ml/min/1.73 m^2^. Similarly, the median UPCR was 0.30 (25^th^ percentile = 0.11, 75^th^ percentile = 0.82) and only 10.1% had nephrotic-range proteinuria (defined by UPCR greater than 2.0). SBP and DBP were above average at the initial visit but gradually decreased over the course of the study (Table 2).

**Table 1:** Characteristics of sample by urinary 8-OHdG and F2-isoprostane predicted mean (95% Confidence Interval) concentrations over time.

	Total (N = 618)	8-OHdG (N = 2,377)^a^ (ng/mg Cr)			F_2_-isoprostane (N = 1,243) (ng/mg Cr)		
	N (%)	Predicted mean (95% CI)			Predicted mean (95% CI)		
All	618 (100)	58.3	55.9	60.8	6.5	5.9	7.2
Sex							
Female	224 (36.3)	60.8	56.7	65.3	6.0	5.0	7.1
Male	394 (63.8)	56.9	53.9	60.0	6.9	6.0	7.8
Race/Ethnicity							
White	360 (58.3)	63.8	60.5	67.3	6.9	6.0	7.9
Black	102 (16.5)	43.7	39.4	48.5*	4.9	3.8	6.4*
Hispanic	85 (13.8)	55.1	49.3	61.6*	6.4	4.9	8.5
Multiracial/Other	71 (11.5)	58.0	51.3	65.5	7.2	5.3	9.8
Age (years) at visit 1							
≤8	171 (27.7)	83.5	78.0	89.3	8.6	7.1	10.4
>8-<13	219 (35.4)	61.1	57.5	64.8*	7.3	6.2	8.7
≥13	228 (36.9)	42.0	39.5	44.6*	4.4	3.7	5.2*
Type of kidney disease							
Non-Glomerular	551 (89.2)	60.1	57.6	62.8	6.8	6.1	7.6
Glomerular	67 (10.8)	43.5	38.0	49.8*	4.2	3.0	6.0*
Urinary cotinine (ng/mL) at visit 1							
<20	516 (96.6)	58.8	56.2	61.5	6.5	5.9	7.3
≥20	18 (3.4)	47.6	37.5	60.5	8.4	4.4	16.1
Body mass index z-score at visit 1							
<0	216 (36.1)	61.1	57.2	65.2	6.8	5.8	8.0
≥0-≤1	193 (32.3)	58.0	55.6	60.5	6.4	5.8	7.1
>1	189 (31.6)	55.1	51.4	59.1*	6.1	5.1	7.2
eGFR (ml/min/1.73 m^2^) at visit 1							
≥90	25 (4.1)	69.3	61.9	77.6	5.1	3.9	6.7
≥60-<90	165 (26.9)	62.8	59.0	66.8	5.9	5.1	6.8
≥30-<60	337 (54.9)	56.9	54.4	59.5	6.8	6.0	7.6
<30	87 (14.2)	51.5	47.4	56.1*	7.8	6.2	9.7
Urinary protein to creatinine ratio (mg/dL:mg/dL) at visit 1							
<0.2	239 (40.3)	57.8	54.2	61.6	6.0	5.2	7.0
≥0.2-≤2	294 (49.6)	58.1	55.4	61.0	6.7	6.0	7.6
>2	60 (10.1)	58.4	52.9	64.6	7.5	5.8	9.8
SBP Z-score at visit 1							
<0	224 (39.1)	56.3	52.5	60.3	6.0	5.1	7.1
≥0	349 (60.9)	60.4	57.1	63.9	6.8	5.9	7.8
DBP Z-score at visit 1							
<0	175 (30.5)	53.2	49.2	57.5	6.0	4.9	7.2
≥0	398 (69.5)	61.4	58.3	64.7*	6.7	5.9	7.6
Hypertensive over follow-up							
No	403 (66.0)	57.2	54.3	60.3	6.1	5.4	7.0
Yes	208 (34.0)	61.0	56.7	65.5	7.3	6.1	8.7
Antihypertensive medication use over follow-up							
No	167 (27.0)	65.7	60.6	71.3	6.3	5.1	7.6
Yes	451 (73.0)	55.8	53.1	58.6*	6.6	5.9	7.5

Abbreviations: 8-OHdG: 8-OH deoxyguanosine; Cr: creatinine; CKD: chronic kidney disease; eGFR: estimated glomerular filtration rate; CI: confidence interval.

^a^There is a discrepancy between the total number of observations in the study (N = 2,464) due to 87 samples missing data on creatinine.

*Significant difference (p < 0.05) in 8-OHdG or F2-isoprostane concentrations in the respective category compared with the reference (the first row listed for each covariate except for body mass index z-score where the reference is ≥0-≤1) from linear mixed models with a random intercept.

**Table 2:** Median (25th, 75th percentile) concentrations of urinary oxidant stress biomarkers, kidney function outcomes, and blood pressure by study visit number.

	Visit 1 (N = 618) Median (25th, 75th percentile)	Visit 2 (N = 572) Median (25th, 75th percentile)	Visit 3 (N = 494) Median (25th, 75th percentile)	Visit 4 (N = 389) Median (25th, 75th percentile)	Visit 5 (N = 255) Median (25th, 75th percentile)	Visit 6 (N = 136) Median (25th, 75th percentile)
Oxidant stress markers						
8-OHdG (ng/mg Cr)	68.8 (38.0, 105.7)	50.6 (34.5, 79.6)	55.2 (32.2, 91.8)	71.0 (43.0, 108.1)	79.8 (54.1, 111.4)	70.1 (47.9, 109.8)
F_2_-isoprostane (ng/mg Cr)^a^	17.2 (1.6, 64.6)	3.6 (1.1, 29.5)	28.6 (4.1, 61.4)	2.7 (1.1, 30.2)	3.0 (1.2, 13.7)	1.5 (0.9, 3.4)
Kidney function outcomes						
eGFR (ml/min/1.73 m^2^)	50.1 (37.6, 63.7)	50.1 (34.1, 63.6)	47.9 (34.2, 62.1)	47.4 (32.2, 60.1)	42.3 (30.4, 56.3)	38.2 (25.9, 53.2)
UPCR (mg/dL:mg/dL)	0.30 (0.11, 0.82)	0.32 (0.12, 1.00)	0.29 (0.11, 0.97)	0.32 (0.12, 0.85)	0.39 (0.14, 1.00)	0.39 (0.11, 1.08)
Blood pressure						
SBP Z-score	0.30 (−0.38, 1.04)	0.26 (−0.44, 0.97)	0.15 (−0.52, 0.98)	0.07 (−0.61, 0.83)	0.04 (−0.63, 0.69)	−0.03 (−0.64, 0.69)
DBP Z-score	0.56 (−0.14, 1.17)	0.35 (−0.25, 1.07)	0.30 (−0.32, 0.95)	0.27 (−0.29, 0.85)	0.22 (−0.46, 0.83)	0.29 (−0.55, 0.94)

Abbreviations: 8-OHdG: 8-OH deoxyguanosine; Cr: creatinine; eGFR: estimated glomerular filtration rate; UPCR: urinary protein to creatinine ratio; SBP: systolic blood pressure; DBP: diastolic blood pressure.

^a^F2-isoprostane was only available in a subset. Counts were as follows: visit 1: N = 173; visit 2: N = 66; visit 3: N = 268; visit 4: N = 388; visit 5: N = 255; visit 6: N = 136.

Urinary concentrations of oxidant stress were associated with selected study participant characteristics. For example, the predicted mean 8-OHdG concentration among Black children was 43.7 ng/mg Cr compared with 63.8 ng/mg Cr among White children (Table 1). Older children (≥13 years) had lower concentrations of both 8-OHdG and F2-isoprostane compared with younger children (≤8 years). Although oxidant stress concentrations were not statistically significantly correlated with urinary cotinine concentrations, those with cotinine values reflecting at least passive smoking (≥20 ng/ml) had greater F2-isoprostane concentrations compared with those with lower cotinine concentrations.

Children with glomerular kidney disease had lower 8-OHdG and F2-isoprostane than those with non-glomerular disease. In bivariate analyses, eGFR was positively associated with 8-OHdG, such that those with the greatest eGFR values had the greatest urinary concentrations of 8-OHdG. However, the opposite was evident for F2-isoprostane: those with lower eGFR had greater urinary concentrations of F2-isoprostane, although the differences were not statistically significant.

Over the course of follow-up, 8-OHdG concentrations had a slow upward trajectory, particularly after the third study visit (Table 2). However, F2-isoprostane concentrations had a spike at the third visit and decreased thereafter. As expected, eGFR gradually decreased and UPCR increased over time. Finally, both SBP and DBP slowly decreased over follow-up.

In adjusted models, baseline 8-OHdG concentrations were positively associated with longitudinal eGFR (Table 3). A log-unit increase in baseline 8-OHdG concentrations was associated with a 5.68 ml/min/1.73 m^2^ increase in eGFR (95% CI: 3.75, 7.61). Similarly, baseline F2-isoprostane concentrations were associated with an increase in eGFR over time (β = 1.73, 95% CI: 0.69, 2.77). These associations did not vary by study visit (Table 4). In addition, baseline 8-OHdG was inversely associated with UPCR, such that a log-unit increase in 8-OHdG was associated with a 21.3% decrease in UPCR (95% CI: 9.5%, 30.9%) (calculated from results in Table 3). Furthermore, the β-coefficient for the association between baseline F2-isoprostane and UPCR was in the same direction but with a wide confidence interval due to smaller sample size and was not statistically significant (β = −0.05, 95% CI: −0.13, 0.02). There was no association between baseline 8-OHdG or F2-isoprostane and either SBP or DBP, and these associations did not vary by study visit (Table 4).

**Table 3:** β-coefficients and 95% confidence intervals from regression models for associations of ln-transformed baseline urinary 8-OHdG and F2-isoprostane concentrations with longitudinal kidney function outcomes and blood pressure.

Kidney function outcomes^a^	8-OHdG	F_2_-isoprostane
	β (95% CI)	β (95% CI)
eGFR (ml/min/1.73 m^2^)	5.68 (3.75, 7.61)	1.73 (0.69, 2.77)
ln(UPCR) (mg/dL:mg/dL)^b^	−0.24 (−0.37, −0.10)	−0.05 (−0.13, 0.02)
Blood pressure^c^		
SBP Z-score	−0.07 (−0.17, 0.03)	−0.03 (−0.08, 0.03)
DBP Z-score	−0.05 (−0.14, 0.03)	−0.02 (−0.07, 0.03)

Abbreviations: 8-OHdG: 8-OH deoxyguanosine; CI: Confidence Interval; eGFR: estimated glomerular filtration rate; UPCR: urinary protein to creatinine ratio; SBP: systolic blood pressure; DBP: diastolic blood pressure.

^a^Models control for visit, urinary creatinine, sex, race/ethnicity, age, glomerular disease type, cotinine, BMI Z-score, and SBP and DBP Z-scores.

^b^UPCR is ln-transformed and thus β-coefficients should be interpreted as follows: a log-unit increase in a given oxidant stress measure is associated with a multiplicative change in UPCR of e^β^.

^c^Models control for visit, urinary creatinine, sex, race/ethnicity, age, glomerular disease type, cotinine, BMI Z-score, and antihypertensive medications.

**Table 4:** β-coefficients and 95% confidence intervals from regression models for associations of ln-transformed baseline urinary 8-OHdG and F2-isoprostane concentrations with longitudinal kidney function outcomes and blood pressure by study visit.

Model^a^	Outcome			
	eGFR	ln(UPCR)	SBP	DBP
	β (95% CI)	β (95% CI)	β (95% CI)	β (95% CI)
8-OHdG				
Visit 1	4.97 (2.95, 7.00)	−0.19 (−0.33, −0.04)	−0.02 (−0.14, 0.10)	−0.03 (−0.14, 0.07)
Visit 2	6.45 (4.39, 8.50)	−0.24 (−0.39, −0.09)	−0.09 (−0.22, 0.03)	0.01 (−0.10, 0.12)
Visit 3	6.04 (3.94, 8.14)	−0.32 (−0.47, −0.16)	−0.07 (−0.20, 0.06)	−0.09 (−0.21, 0.03)
Visit 4	5.89 (3.76, 8.02)	−0.21 (−0.37, −0.06)	−0.08 (−0.22, 0.06)	−0.12 (−0.24, 0.01)
Visit 5	5.54 (3.26, 7.81)	−0.23 (−0.40, −0.05)	−0.08 (−0.24, 0.09)	−0.05 (−0.19, 0.10)
Visit 6	5.84 (3.44, 8.23)	−0.26 (−0.44, −0.08)	−0.18 (−0.36, 0.00)	−0.09 (−0.26, 0.07)
F_2_-isoprostane				
Visit 1	1.28 (0.03, 2.53)	0.00 (−0.09, 0.10)	−0.03 (−0.12, 0.07)	−0.03 (−0.11, 0.06)
Visit 2	4.97 (2.68, 7.27)	−0.09 (−0.30, 0.11)	−0.16 (−0.38, 0.06)	−0.10 (−0.31, 0.11)
Visit 3	1.50 (0.32, 2.69)	−0.08 (−0.17, 0.02)	−0.01 (−0.10, 0.07)	−0.01 (−0.09, 0.06)
Visit 4	1.83 (0.71, 2.95)	−0.04 (−0.13, 0.04)	−0.02 (−0.09, 0.05)	−0.05 (−0.11, 0.02)
Visit 5	1.77 (0.59, 2.95)	−0.06 (−0.15, 0.03)	−0.08 (−0.16, 0.00)	−0.01 (−0.09, 0.06)
Visit 6	2.09 (0.68, 3.49)	−0.13 (−0.25, −0.02)	−0.02 (−0.14, 0.09)	0.03 (−0.07, 0.14)

Abbreviations: CI: Confidence Interval; eGFR: estimated glomerular filtration rate; UPCR: urinary protein to creatinine ratio; SBP: systolic blood pressure; DBP: diastolic blood pressure.

^a^Models control for visit, creatinine, sex, race/ethnicity, age, glomerular disease type, cotinine, BMI Z-score, in eGFR and UPCR models, SBP and DBP; in SBP and DBP models, antihypertensive medications.

The association between longitudinal 8-OHdG concentrations and longitudinal eGFR was also positive, but smaller in magnitude than that with baseline levels (Table 5). A log-unit increase in 8-OHdG was associated with a 0.81 ml/min/1.73 m^2^ increase in eGFR (95% Confidence Interval (CI): 0.22, 1.39). When allowed to vary over time, this association increased over time and was strongest at the fourth, fifth, and sixth visits compared with the first three (Table 6). However, there was no association with longitudinal F2-isoprostane and eGFR over time, or between either measure of oxidant stress and UPCR or blood pressure over time (Table 5). These associations did not vary by study visit (Table 6).

**Table 5:** β-coefficients and 95% confidence intervals from regression models for associations of ln-transformed longitudinal urinary 8-OHdG and F2-isoprostane concentrations with longitudinal kidney function outcomes and blood pressure.

Kidney function outcomes^a^	8-OHdG	F_2_-isoprostane
	β (95% CI)	β (95% CI)
eGFR (ml/min/1.73 m^2^)	0.81 (0.22, 1.39)	0.01 (−0.33, 0.36)
ln(UPCR) (mg/dL:mg/dL)^b^	0.00 (−0.05, 0.05)	0.02 (−0.01, 0.05)
Blood pressure^c^		
SBP Z-score	−0.03 (−0.09, 0.03)	−0.01 (−0.04, 0.03)
DBP Z-score	−0.02 (−0.07, 0.04)	−0.01 (−0.04, 0.02)

Abbreviations: 8-OHdG: 8-OH deoxyguanosine; CI: Confidence Interval; eGFR: estimated glomerular filtration rate; UPCR: urinary protein to creatinine ratio; SBP: systolic blood pressure; DBP: diastolic blood pressure.

^a^Models control for visit, urinary creatinine, sex, race/ethnicity, age, glomerular disease type, cotinine, BMI Z-score, and SBP and DBP Z-scores.

^b^UPCR is ln-transformed and thus β-coefficients should be interpreted as follows: a log-unit increase in a given oxidant stress measure is associated with a multiplicative change in UPCR of e^β^.

^c^Models control for visit, urinary creatinine, sex, race/ethnicity, age, glomerular disease type, cotinine, BMI Z-score, and antihypertensive medications.

**Table 6:** β-coefficients and 95% confidence intervals from regression models for associations of ln-transformed longitudinal urinary 8-OHdG and F2-isoprostane concentrations with longitudinal kidney function outcomes and blood pressure by study visit.

Expsure^a^	Outcome			
	eGFR	ln(UPCR)	SBP	DBP
	β (95% CI)	β (95% CI)	β (95% CI)	β (95% CI)
8-OHdG				
Visit 1	−0.14 (−1.09, 0.81)	0.01 (−0.07, 0.09)	0.04 (−0.05, 0.14)	0.01 (−0.08, 0.10)
Visit 2	0.51 (−0.73, 1.74)	0.02 (−0.09, 0.12)	−0.03 (−0.15, 0.10)	0.00 (−0.12, 0.12)
Visit 3	0.64 (−0.40, 1.69)	0.02 (−0.07, 0.11)	0.01 (−0.10, 0.12)	0.06 (−0.04, 0.16)
Visit 4	1.64 (0.49, 2.80)	0.04 (−0.06, 0.14)	−0.15 (−0.27, −0.03)	−0.11 (−0.23, 0.00)
Visit 5	1.89 (0.30, 3.48)	−0.06 (−0.19, 0.08)	−0.08 (−0.25, 0.08)	−0.09 (−0.25, 0.06)
Visit 6	1.69 (0.11, 3.28)	−0.12 (−0.26, 0.01)	−0.05 (−0.22, 0.11)	−0.06 (−0.21, 0.10)
F_2_-isoprostane				
Visit 1	−0.46 (−1.31, 0.39)	0.05 (−0.02, 0.12)	0.00 (−0.09, 0.09)	−0.01 (−0.09, 0.07)
Visit 2	−0.18 (−2.09, 1.72)	−0.03 (−0.19, 0.14)	0.18 (−0.02, 0.37)	−0.16 (−0.34, 0.02)
Visit 3	−0.09 (−0.96, 0.79)	0.03 (−0.04, 0.11)	0.01 (−0.08, 0.10)	−0.01 (−0.09, 0.08)
Visit 4	0.35 (−0.25, 0.94)	0.00 (−0.05, 0.05)	−0.03 (−0.09, 0.03)	−0.02 (−0.07, 0.04)
Visit 5	−0.05 (−0.84, 0.74)	0.02 (−0.04, 0.09)	−0.02 (−0.10, 0.07)	0.03 (−0.05, 0.11)
Visit 6	0.03 (−0.94, 1.00)	−0.02 (−0.10, 0.06)	0.00 (−0.10, 0.10)	0.00 (−0.09, 0.10)

Abbreviations: 8-OHdG: 8-OH deoxyguanosine; CI: Confidence Interval; eGFR: estimated glomerular filtration rate; UPCR: urinary protein to creatinine ratio; SBP: systolic blood pressure; DBP: diastolic blood pressure.

^a^Models control for visit, creatinine, sex, race/ethnicity, age, glomerular disease type, cotinine, BMI Z-score, in eGFR and UPCR models, SBP and DBP; in SBP and DBP models, antihypertensive medications.

Urinary concentrations of both 8-OHdG and F2-isoprostane were associated with a longer time to RRT or a reduced hazard of RRT (Table 7). A log-unit increase in 8-OHdG was associated with a decreased hazard of RRT, which was stronger when modeled over time (0.68, 95% CI: 0.49, 0.96) than at baseline (HR = 0.87, 95% CI: 0.72, 1.07). The same pattern was observed for F2-isoprostane, but the estimate for time-varying F2-isoprostane had poor precision (HR = 0.66, 95% CI: 0.31, 1.41).

**Table 7:** Adjusted Hazard Ratios (HR)a and 95% Confidence Intervals (CI) from Cox Proportional Hazards Models for the associations of ln-transformed urinary 8-OHdG and F2-isoprostane concentrations with time to renal replacement therapy and/or end-stage renal disease.

	N (%)^a^ events	HR (95% CI)	HR (95% CI)
		*Baseline oxidant stress*	*Time-varying oxidant stress*
8-OHdG	179 (33.3%)	0.87 (0.72, 1.07)	0.68 (0.49, 0.96)
F_2_-isoprostane	42 (28.4%)	0.74 (0.59, 0.92)	0.66 (0.31, 1.41)

Abbreviations: 8-OHdG: 8-OH deoxyguanosine; HR: hazard ratio; CI: Confidence Interval.

^a^Models control for creatinine, sex, race/ethnicity, age, glomerular disease type, cotinine, BMI Z-score, SBP and DBP.

^b^Denominator = 538 individuals with baseline samples for 8-OHdG and N = 148 individuals with baseline samples for F2-isoprostane.

Sensitivity analyses yielded largely consistent results with the primary analyses. First, controlling for eCER instead of urinary creatinine in all models produced almost identical results (data not shown). Second, models with baseline measures of oxidant stress in relation to longitudinal outcomes starting at the second visit were very similar to the results from models with baseline oxidant stress and all outcomes over time, as shown in Table 5. Third, when models were stratified by eGFR category and median UPCR, the directions of the associations persisted compared with the results from the original models, but the magnitude and statistical significance attenuated for some (Supplemental Tables 1 and 2). For example, in models for the association between baseline 8-OHdG and longitudinal eGFR, the measure of association for a log-unit increase in 8-OHdG was an order of magnitude smaller among those with eGFR <45 ml/min/1.73 m^2^ (β = 0.84, 95% CI: −0.76, 2.43) than among those with eGFR ≥45 ml/min/1.73 m^2^ (β = 4.88, 95% CI: 2.79, 6.97). A similar pattern was noted across models for the association between longitudinal 8-OHdG and longitudinal eGFR, with the result among those with eGFR <45 ml/min/1.73 m^2^ (β = 0.56, 95% CI: −0.16, 1.28) being slightly less than that among those with eGFR ≥45 ml/min/1.73 m^2^ (β = 0.85, 95% CI: 0.05, 1.65). Patterns were consistent across UPCR strata (Supplemental Table 2). Finally, restricting the study sample to a balanced dataset (i.e., three visits per participant) produced similar results as well (data not shown).

## Discussion

In this cohort of children with CKD, two measures of oxidant stress, 8-OHdG and F2-isoprostane, were evaluated in serial urine samples collected over time throughout the course of kidney function decline. To our knowledge, this is the first prospective observational cohort study to evaluate the association between oxidant stress and measures of kidney function over time in a CKD population and, specifically, among children. We found a positive association between 8-OHdG, considered both as a baseline measure and longitudinally as repeated measures, and eGFR over time. The association was an order of magnitude greater when baseline concentrations were related to eGFR over time. Baseline F2-isoprostane was also associated with greater eGFR over time. In addition, baseline, but not longitudinal 8-OHdG was associated with lower UPCR over time. Finally, 8-OHdG and F2-isoprostane were associated with a reduced hazard of RRT. These results were robust to alternative methods of controlling for urinary creatinine, modeling baseline oxidant stress in relation to later outcomes over time, and limiting the dataset so that all participants had an equal number of observations over time.

Several studies have documented increased levels of oxidant stress among CKD patients compared with healthy controls^13,14,40^, and associations between oxidant stress and advanced stages of CKD and reduced eGFR^14,15^. These four studies assessed oxidant stress using a variety of indicator molecules in the plasma while our study relied on assays of urinary excretion of 8-OHdG and F_2_-isoprostane. One of the only longitudinal studies compared measures of oxidant stress among those with kidney failure before and after transplantation and found that levels of F_2_-isprostane were significantly reduced one week after transplant to the levels of healthy subjects, which persisted up to two months after transplant^41^. In contrast, we found positive associations between urinary oxidant stress biomarkers and kidney function, which was unexpected. Specifically, we found that greater urinary concentrations of oxidant stress biomarkers at baseline were related to higher eGFRs,reduced UPCR over time, and a reduced hazard of RRT; and taken together, a better renal function profile.

There were several key differences between previous studies and ours. First, our study was conducted among children, the majority of whom had non-glomerular disease, in whom this relationship has seldom been examined. Second, as mentioned above, we measured urinary oxidant stress biomarkers whereas other studies used plasma as their matrix. This is important because kidney function directly impacts the ability to filter and excrete solutes such as the oxidant stress biomarkers^29^. Therefore, it is possible that those with diminished kidney function were the least able to filter the biomarkers at the glomerular level and/or secrete oxidant stress analytes into the urine. These disturbances would result in low urinary concentrations. This is referred to in the epidemiologic literature as reverse causation, in that kidney function may affect the exposure measures of interest^42,43^. Pharmacokinetic differences between people, such as in their ability to filter and excrete oxidant stress measures or other compounds such as environmental toxicants, can affect the exposure assessment; in this case, concentrations in urine^44,45^. Furthermore, the possibility of ‘reverse causation’ could be amplified in a cross-sectional study where oxidant stress and eGFR were assessed at the same time. This is consistent with our observation that the associations were stronger when we considered baseline oxidant stress in relation to longitudinal outcomes compared with the analysis of longitudinal oxidant stress measures with longitudinal outcomes. Cross-sectional studies that have measured urinary F_2_-isoprostanes have reported positive associations with eGFR^46–48^, and one also found a negative association with albuminuria. Our results provide evidence of this same phenomenon even in a longitudinal setting, and is supported by our consistent observation that urinary oxidant stress biomarkers were associated with a reduced hazard of RRT. Another recent longitudinal study found no association between baseline oxidant stress markers and GFR, but a significant association with increased albuminuria^49^. None of these reports documented a lower GFR in association with graded increases in excretion of oxidant stress biomarkers.

In an effort to better understand the possibility of this ‘reverse causation’, we stratified models by eGFR category as well as median UPCR. We hypothesized that if reduced excretion of urinary biomarkers of oxidant stress was due to poor kidney function (i.e., low eGFR), the positive association between oxidant stress biomarkers and eGFR would be attenuated or even disappear in results from stratified models in which comparisons were being made among individuals with more similar kidney function. In these sensitivity analyses, we found that 8-OHdG was still positively associated with eGFR, but with a smaller magnitude among those with eGFR <45 ml/min/1.73 m^2^, and no longer statistically significant. However, the associations remained essentially unchanged among those with eGFR ≥45 ml/min/1.73 m^2^. These results are consistent with our hypothesis that kidney function influences urinary excretion of oxidant stress biomarkers (i.e., reverse causation). The persistent positive association for those with eGFR ≥45 ml/min/1.73 m^2^, is likely due to the substantial degree of variation in kidney function and excretory capability in this group despite the stratification.

This study benefited from several strengths. The CKiD study has a large biorepository from a unique population of children with CKD followed over time. This allowed us to test the hypothesis with a longitudinal design, which has seldom been done. Second, due to the large number of samples on each study participant, we were able to conduct several types of analysis. We examined longitudinal oxidant stress in relation to longitudinal kidney function and blood pressure over time; as well as baseline levels of oxidant stress in relation to these same longitudinal outcomes. This enabled us to better understand the dynamics of this complex relationship.

However, this study had several limitations. First, although measuring urinary biomarkers of oxidant stress among a CKD population is problematic, it has been suggested that urine is a superior matrix for measuring F2-isoprostane compared with plasma^50^. In addition, although the samples were stored for variable periods of time, urine specimens kept at −80 °C for over a decade have been utilized in previous studies of oxidant stress in CKD^46,47,49^. Further, it is important to note that there are no data about protein binding and tubular handling of 8-OHdG or F_2_-isoprostane. This limits our ability to attribute the changes in oxidant stress biomarker excretion to specific aspects of kidney function. Measuring analytes in serum or plasma among CKD populations may not obviate this complication because blood levels may be elevated due to altered distribution in body compartments and decreased clearance^29^, which would lead to the same problem, but in the opposite direction. Further investigation of this issue is necessary in order to better understand if this phenomenon may be underlying the previous observations that blood levels of oxidant stress are inversely related to eGFR^14,15^. Another limitation was that this study consisted of prevalent CKD patients at different stages, which may obscure the ability to infer the directionality of the relationship between oxidant stress and kidney function. Although it is acknowledged that CKD stimulates oxidant stress through mechanisms such as tubular production of oxygenated free radicals which also influences disease progression^11^, the directionality of this relationship has been difficult to determine due to the cross-sectional design in prior studies^11^. In addition, we were unable to distinguish between oxidant stress produced as part of the kidney injury process versus exposure to exogenous chemicals and drugs. Our findings do not directly address the question of the contribution of oxidant stress in promoting CKD progression. They do raise questions regarding the assessment of this injury process based on urinary excretion of oxidant stress biomarkers.

## Conclusions

In this longitudinal investigation of oxidant stress measures in relation to kidney function over time among children with CKD, we observed that urinary excretion of oxidant stress biomarkers was associated with increased eGFR, decreased proteinuria, and a reduced risk of RRT, an improved renal function profile overall. No associations were observed with blood pressure. Although increased oxidant stress contributes to progressive renal injury in patients with CKD, our findings suggest that monitoring the process using urinary levels of oxidant stress biomarkers is more a reflection of the current level of kidney function, glomerfular and/or tubular, rather than the trajectory of disease progression.

## Supplementary information

Supplementary information.

## References

[1] Levey AS, Coresh J (2012). Chronic kidney disease. The lancet.

[2] Coresh J, Astor BC, Greene T, Eknoyan G, Levey AS (2003). Prevalence of chronic kidney disease and decreased kidney function in the adult US population: Third national health and nutrition examination survey. American Journal of Kidney Diseases.

[3] Levey AS (2003). National Kidney Foundation practice guidelines for chronic kidney disease: evaluation, classification, and stratification. Annals of internal medicine.

[4] Hoerger TJ (2015). The future burden of CKD in the United States: a simulation model for the CDC CKD Initiative. American Journal of Kidney Diseases.

[5] Wong CJ, Moxey-Mims M, Jerry-Fluker J, Warady BA, Furth SL (2012). CKiD (CKD in children) prospective cohort study: a review of current findings. American journal of kidney diseases: the official journal of the National Kidney Foundation.

[6] Becherucci F, Roperto RM, Materassi M, Romagnani P (2016). Chronic kidney disease in children. Clinical kidney journal.

[7] Staples A, Wong C (2010). Risk factors for progression of chronic kidney disease. Current opinion in pediatrics.

[8] Small DM, Coombes JS, Bennett N, Johnson DW, Gobe GC (2012). Oxidative stress, anti‐oxidant therapies and chronic kidney disease. Nephrology.

[9] Okamura DM, Himmelfarb J (2009). Tipping the redox balance of oxidative stress in fibrogenic pathways in chronic kidney disease. Pediatric nephrology.

[10] Schrier RW, Harris DC, Chan L, Shapiro JI, Caramelo C (1988). Tubular hypermetabolism as a factor in the progression of chronic renal failure. American journal of kidney diseases: the official journal of the National Kidney Foundation.

[11] Vaziri ND, Rodríguez-Iturbe B (2006). Mechanisms of disease: oxidative stress and inflammation in the pathogenesis of hypertension. Nature Reviews Nephrology.

[12] Li L (2018). High Salt Enhances Reactive Oxygen Species and Angiotensin II Contractions of Glomerular Afferent Arterioles From Mice With Reduced Renal Mass. Hypertension.

[13] Oberg BP (2004). Increased prevalence of oxidant stress and inflammation in patients with moderate to severe chronic kidney disease. Kidney international.

[14] Karamouzis I (2008). Increase in oxidative stress but not in antioxidant capacity with advancing stages of chronic kidney disease. American journal of nephrology.

[15] Dounousi E (2006). Oxidative stress is progressively enhanced with advancing stages of CKD. American Journal of Kidney Diseases.

[16] Milne GL, Musiek ES, Morrow JD (2005). F2-isoprostanes as markers of oxidative stress *in vivo*: an overview. Biomarkers: biochemical indicators of exposure, response, and susceptibility to chemicals.

[17] Roberts LJ, Morrow JD (2000). Measurement of F2-isoprostanes as an index of oxidative stress *in vivo*. Free Radical Biology and Medicine.

[18] Wu LL, Chiou C-C, Chang P-Y, Wu JT (2004). Urinary 8-OHdG: a marker of oxidative stress to DNA and a risk factor for cancer, atherosclerosis and diabetics. Clinica Chimica Acta.

[19] Halliwell B, Whiteman M (2004). Measuring reactive species and oxidative damage *in vivo* and in cell culture: how should you do it and what do the results mean?. British journal of pharmacology.

[20] Furth SL (2006). Design and methods of the Chronic Kidney Disease in Children (CKiD) prospective cohort study. Clinical Journal of the American Society of Nephrology.

[21] Hornung RW, Reed LD (1990). Estimation of average concentration in the presence of nondetectable values. Applied occupational and environmental hygiene.

[22] Wong CS (2009). Association of proteinuria with race, cause of chronic kidney disease, and glomerular filtration rate in the chronic kidney disease in children study. Clinical Journal of the American Society of Nephrology.

[23] Schwartz GJ (2009). New equations to estimate GFR in children with CKD. Journal of the American Society of Nephrology: JASN.

[24] Flynn JT (2008). Blood pressure in children with chronic kidney disease: a report from the Chronic Kidney Disease in Children study. Hypertension (Dallas, Tex.: 1979).

[25] National High Blood Pressure Education Program Working Group on High Blood Pressure in Children and Adolescents (2004). The fourth report on the diagnosis, evaluation, and treatment of high blood pressure in children and adolescents. Pediatrics.

[26] Warady BA (2015). Predictors of Rapid Progression of Glomerular and Nonglomerular Kidney Disease in Children and Adolescents: The Chronic Kidney Disease in Children (CKiD) Cohort. American journal of kidney diseases: the official journal of the National Kidney Foundation.

[27] Kleinbaum, D. & Klein, M. J. N. Y. Springer-Verlag. Survival Analysis: A self-learning text, 2005 (2011).

[28] Barr DB (2004). Urinary creatinine concentrations in the US population: implications for urinary biologic monitoring measurements. Environmental health perspectives.

[29] Weaver VM, Kotchmar DJ, Fadrowski JJ, Silbergeld EK (2016). Challenges for environmental epidemiology research: are biomarker concentrations altered by kidney function or urine concentration adjustment?. Journal of Exposure Science and Environmental Epidemiology.

[30] Wright J (2002). Effect of Blood Pressure Lowering and Antihypertensive Drug Class on Progression of Hypertensive Kidney DiseaseResults From the AASK Trial. JAMA.

[31] Keaney JF (2003). Obesity and systemic oxidative stress: clinical correlates of oxidative stress in the Framingham Study. Arteriosclerosis, thrombosis, and vascular biology.

[32] Tamura S (2006). Evaluation of a urinary multi-parameter biomarker set for oxidative stress in children, adolescents and young adults. Free radical research.

[33] Kosecik M, Erel O, Sevinc E, Selek S (2005). Increased oxidative stress in children exposed to passive smoking. International journal of cardiology.

[34] Fathallah-Shaykh SA (2015). Progression of Pediatric CKD of Nonglomerular Origin in the CKiD Cohort. Clinical Journal of the American Society of Nephrology.

[35] Feairheller DL (2011). Racial differences in oxidative stress and inflammation: *in vitro* and *in vivo*. Clinical and translational science.

[36] Luo S (2019). Serum Metabolomic Alterations Associated with Proteinuria in CKD. Clinical Journal of the American Society of Nephrology.

[37] Abdelmalek JA, Gansevoort RT, Heerspink HJL, Ix JH, Rifkin DE (2014). Estimated albumin excretion rate versus urine albumin-creatinine ratio for the assessment of albuminuria: a diagnostic test study from the Prevention of Renal and Vascular Endstage Disease (PREVEND) Study. American Journal of Kidney Diseases.

[38] Ix JH (2011). Equations to estimate creatinine excretion rate: the CKD epidemiology collaboration. Clinical Journal of the American Society of Nephrology.

[39] [No authors listed]. Chapter 1: Definition and classification of CKD. *Kidney international supplements* **3**, 19–62, 10.1038/kisup.2012.64 (2013). 10.1038/kisup.2012.64 PMC4089693 25018975

[40] Himmelfarb J (2004). Oxidative stress is increased in critically ill patients with acute renal failure. Journal of the American Society of Nephrology.

[41] Simmons EM (2005). Effect of renal transplantation on biomarkers of inflammation and oxidative stress in end-stage renal disease patients. Transplantation.

[42] Dhingra R, Winquist A, Darrow LA, Klein M, Steenland K (2016). A study of reverse causation: Examining the associations of perfluorooctanoic acid serum levels with two outcomes. Environmental health perspectives.

[43] Watkins DJ (2013). Exposure to perfluoroalkyl acids and markers of kidney function among children and adolescents living near a chemical plant. Environmental health perspectives.

[44] Longnecker MP (2006). Pharmacokinetic variability and the miracle of modern analytical chemistry. Epidemiology.

[45] Dorne J, Renwick AG (2005). The refinement of uncertainty/safety factors in risk assessment by the incorporation of data on toxicokinetic variability in humans. Toxicological Sciences.

[46] Nerpin, E. *et al*. Inflammation, oxidative stress, glomerular filtration rate, and albuminuria in elderly men: a cross-sectional study. **5**, 537, 10.1186/1756-0500-5-537 (2012). 10.1186/1756-0500-5-537 PMC3527356 23016573

[47] Upadhyay A (2011). Inflammation, kidney function and albuminuria in the Framingham Offspring cohort. Nephrology, dialysis, transplantation: official publication of the European Dialysis and Transplant Association - European Renal Association.

[48] Sauriasari R, Wulandari F, Nurifahmi R, Sekar AP, Susilo VY (2018). The Correlation Between Urinary 8-Iso-Prostaglandin F2alpha and Hydrogen Peroxide Toward Renal Function in T2DM Patients Consuming Sulfonylurea and Combination of Metformin-Sulfonylurea. Current diabetes reviews.

[49] Schei J (2018). Urinary Markers of Oxidative Stress Are Associated With Albuminuria But Not GFR Decline. Kidney International Reports.

[50] Nourooz-Zadeh Jaffar (2008). Key issues in F2-isoprostane analysis. Biochemical Society Transactions.

