# Supplementary material for: Oxidant stress and renal function among children with chronic kidney disease: a repeated measures study
Source: Sci Rep. 2020 Feb 21;10:3129. doi: 10.1038/s41598-020-59962-9 (PMC7035390; doi:10.1038/s41598-020-59962-9)
Supplement: Supplementary file 1 — Supplementary information. [file 41598_2020_59962_MOESM1_ESM.docx]

**SUPPLEMENTAL TABLES**

**Oxidant stress and renal function among children with chronic kidney disease: a repeated measures study**

Melanie H. Jacobson PhD MPH^1^, Mengling Liu PhD^2^, Yinxiang Wu MA^2^, Susan Furth MD, PhD^3^, Bradley Warady MD^4^, Howard Trachtman MD^5*^, Leonardo Trasande MD MPP ^1,2,6,7^

^1^ Department of Pediatrics, Division of Environmental Pediatrics, NYU Langone Medical Center, New York, NY, USA

^2^ Departments of Population Health and Environmental Medicine, NYU Langone Medical Center, New York, NY, USA

^3^ Division of Nephrology, Department of Pediatrics, Children’s Hospital of Philadelphia, Philadelphia, PA, USA

^4^ Division of Nephrology, Department of Pediatrics, Children's Mercy Kansas City, Kansas City, MO, USA

^5^ Department of Pediatrics, Division of Nephrology, NYU Langone Medical Center, New York, NY, USA

^6^ NYU Wagner School of Public Service, New York, NY, USA

^7^ NYU College of Global Public Health, New York, NY, USA

| **Supplemental Table 1.** β-coefficients and 95% confidence intervals from regression models for associations of ln-transformed urinary 8-OHdG and F_2_-isoprostane concentrations with longitudinal kidney function outcomes and blood pressure stratified by eGFR category at 45 ml/min/1.73 m^2^ | | | | | | | | | | | | | |
| --- | --- | --- | --- | --- | --- | --- | --- | --- | --- | --- | --- | --- | --- |
|  | **8-OHdG**^a^ | | | | | |  | **F_2_-isoprostane**^b^ | | | | | |
|  | **eGFR ≥45 ml/min/1.73 m^2^** | | | **eGFR <45 ml/min/1.73 m^2^** | | |  | **eGFR ≥45 ml/min/1.73 m^2^** | | | **eGFR <45 ml/min/1.73 m^2^** | | |
|  | β | (95% CI) | | β | (95% CI) | |  | β | (95% CI) | | β | (95% CI) | |
|  | | | | |  |  |  |  |  |  |  |  |  |
| ***Baseline oxidative stress and longitudinal outcomes*** | | | | |  |  |  |  |  |  |  |  |  |
| Kidney function outcomes^c^ |  |  |  |  |  |  |  |  |  |  |  |  |  |
| eGFR (ml/min/1.73 m^2^) | 4.88 | 2.79 | 6.97 | 0.84 | -0.76 | 2.43 |  | 0.61 | -0.49 | 1.71 | 0.24 | -0.93 | 1.41 |
| ln(UPCR) (mg/dL:mg/dL)^d^ | -0.09 | -0.26 | 0.08 | -0.15 | -0.37 | 0.07 |  | 0.00 | -0.09 | 0.08 | -0.04 | -0.19 | 0.11 |
| Blood pressure^e^ |  |  |  |  |  |  |  |  |  |  |  |  |  |
| SBP Z-score | -0.11 | -0.23 | 0.01 | 0.00 | -0.19 | 0.19 |  | -0.02 | -0.08 | 0.04 | -0.08 | -0.21 | 0.05 |
| DBP Z-score | -0.06 | -0.16 | 0.05 | 0.00 | -0.15 | 0.14 |  | -0.01 | -0.07 | 0.04 | -0.02 | -0.14 | 0.10 |
|  | | | | | | | | | | | | | |
| ***Longitudinal oxidative stress and longitudinal outcomes*** | | | | | | | | | | | | | |
| Kidney function outcomes^c^ |  | | |  | | |  |  | | |  | | |
| eGFR (ml/min/1.73 m^2^) | 0.85 | 0.05 | 1.65 | 0.56 | -0.16 | 1.28 |  | -0.15 | -0.63 | 0.32 | 0.26 | -0.18 | 0.70 |
| ln(UPCR) (mg/dL:mg/dL)^d^ | 0.03 | -0.03 | 0.10 | -0.02 | -0.09 | 0.06 |  | 0.03 | 0.00 | 0.07 | -0.03 | -0.08 | 0.02 |
| Blood pressure^e^ |  |  |  |  |  |  |  |  |  |  |  |  |  |
| SBP Z-score | -0.02 | -0.09 | 0.05 | -0.02 | -0.12 | 0.08 |  | 0.01 | -0.03 | 0.06 | -0.02 | -0.08 | 0.05 |
| DBP Z-score | -0.01 | -0.07 | 0.06 | -0.01 | -0.10 | 0.09 |  | -0.01 | -0.05 | 0.03 | 0.00 | -0.06 | 0.06 |
| ^a^Among those with eGFR ≥45 ml/min/1.73 m^2^, n=370 individuals, 1526 observations; among those with eGFR <45 ml/min/1.73 m^2^, 244 individuals, 927 observations | | | | | | | | | | | | | |
| ^b^Among those with eGFR ≥45 ml/min/1.73 m^2^, N=336 individuals, 854 observations; among those with eGFR <45 ml/min/1.73 m^2^, 182 individuals, 426 observations | | | | | | | | | | | | | |
| ^c^Models control for visit, urinary creatinine, sex, race/ethnicity, age, glomerular disease type, cotinine, BMI Z-score, and SBP and DBP Z-scores | | | | | | | | | | | | | |
| ^d^UPCR is ln-transformed and thus β-coefficients should be interpreted as follows: a log-unit increase in a given oxidative stress measure is associated with a multiplicative change in UPCR of e^β^ | | | | | | | | | | | | | |
| ^e^Models control for visit, urinary creatinine, sex, race/ethnicity, age, glomerular disease type, cotinine, BMI Z-score, and antihypertensive medications | | | | | | | | | | | | | |

| **Supplemental Table 2.** β-coefficients and 95% confidence intervals from regression models for associations of ln-transformed urinary 8-OHdG and F_2_-isoprostane concentrations with longitudinal kidney function outcomes and blood pressure stratified by median baseline UPCR (0.30 mg/dL: mg/dL) | | | | | | | | | | | | | |
| --- | --- | --- | --- | --- | --- | --- | --- | --- | --- | --- | --- | --- | --- |
|  | **8-OHdG**^a^ | | | | | |  | **F_2_-isoprostane**^b^ | | | | | |
|  | **UPCR ≥0.30 mg/dL:mg/dL** | | | **UPCR < 0.30 mg/dL:mg/dL** | | |  | **UPCR ≥0.30 mg/dL:mg/dL** | | | **UPCR < 0.30 mg/dL:mg/dL** | | |
|  | β | (95% CI) | | β | (95% CI) | |  | β | (95% CI) | | β | (95% CI) | |
|  | | | | |  |  |  |  |  |  |  |  |  |
| ***Baseline oxidative stress and longitudinal outcomes*** | | | | |  |  |  |  |  |  |  |  |  |
| Kidney function outcomes^c^ |  |  |  |  |  |  |  |  |  |  |  |  |  |
| eGFR (ml/min/1.73 m^2^) | 4.74 | 2.23 | 7.25 | 5.08 | 2.41 | 7.75 |  | 1.46 | 0.05 | 2.88 | 1.77 | 0.39 | 3.15 |
| ln(UPCR) (mg/dL:mg/dL)^d^ | -0.04 | -0.19 | 0.11 | -0.23 | -0.35 | -0.11 |  | -0.02 | -0.12 | 0.07 | -0.05 | -0.13 | 0.02 |
| Blood pressure^e^ |  |  |  |  |  |  |  |  |  |  |  |  |  |
| SBP Z-score | -0.03 | -0.18 | 0.11 | -0.12 | -0.26 | 0.02 |  | -0.06 | -0.15 | 0.03 | 0.00 | -0.08 | 0.07 |
| DBP Z-score | -0.07 | -0.19 | 0.05 | -0.02 | -0.13 | 0.10 |  | -0.04 | -0.11 | 0.04 | 0.00 | -0.07 | 0.06 |
|  | | | | | | | | | | | | | |
| ***Longitudinal oxidative stress and longitudinal outcomes*** | | | | | | | | | | | | | |
| Kidney function outcomes^c^ |  | | |  | | |  |  | | |  | | |
| eGFR (ml/min/1.73 m^2^) | 1.11 | 0.22 | 1.99 | 0.50 | -0.29 | 1.28 |  | 0.10 | -0.47 | 0.66 | 0.06 | -0.38 | 0.50 |
| ln(UPCR) (mg/dL:mg/dL)^d^ | 0.00 | -0.07 | 0.07 | 0.01 | -0.05 | 0.07 |  | 0.02 | -0.02 | 0.06 | 0.02 | -0.02 | 0.06 |
| Blood pressure^e^ |  |  |  |  |  |  |  |  |  |  |  |  |  |
| SBP Z-score | -0.05 | -0.14 | 0.04 | -0.01 | -0.08 | 0.07 |  | -0.01 | -0.07 | 0.04 | -0.01 | -0.06 | 0.03 |
| DBP Z-score | -0.02 | -0.11 | 0.06 | 0.00 | -0.07 | 0.07 |  | -0.01 | -0.06 | 0.04 | -0.03 | -0.07 | 0.02 |

| ^a^Among those with UPCR≥0.30 mg/dL:mg/dL, n=298 individuals, 1152 observations; among those with UPCR<0.30 mg/dL: mg/dL, 295 individuals, 1232 observations |
| --- |
| ^b^Among those with UPCR≥0.30 mg/dL:mg/dL, N=234 individuals, 567 observations; among those with UPCR<0.30 mg/dL: mg/dL, 269 individuals, 677 observations |
| ^c^Models control for visit, urinary creatinine, sex, race/ethnicity, age, glomerular disease type, cotinine, BMI Z-score, and SBP and DBP Z-scores |
| ^d^UPCR is ln-transformed and thus β-coefficients should be interpreted as follows: a log-unit increase in a given oxidative stress measure is associated with a multiplicative change in UPCR of e^β^ |
| ^e^Models control for visit, urinary creatinine, sex, race/ethnicity, age, glomerular disease type, cotinine, BMI Z-score, and antihypertensive medications |
